# Life-threatening upper gastrointestinal bleeding caused by pancreatic pseudocysts: A case report

**DOI:** 10.1097/MD.0000000000046917

**Published:** 2026-01-16

**Authors:** Shifeng Teng, Minghao Huang

**Affiliations:** a Department of General Surgery, Tongji Hospital, School of Medicine, Tongji University, Shanghai, China; b School of Medicine, Tongji University, Shanghai, China.

**Keywords:** gastrointestinal hemorrhage, general surgery, melena, pancreatic pseudocyst

## Abstract

**Rationale:**

Pancreatic pseudocysts are fluid-filled sacs that typically develop as a complication of pancreatitis or abdominal trauma. These lesions are relatively rare, with an estimated incidence of 0.5 to 1 case per 100,000 people annually. While asymptomatic pseudocysts often resolve with conservative management, symptomatic cases or those complicated by infection, hemorrhage, or obstruction generally require surgical intervention.

**Patient concerns:**

A 45-year-old man had recurrent black stools and vomiting blood. Initially diagnosed with gastritis, his condition worsened despite treatment, leading to severe bleeding.

**Diagnoses:**

Imaging showed a suspected stomach hematoma, but the cause was a pancreatic pseudocyst eroding the stomach wall and a gastric artery.

**Interventions:**

Emergency surgery.

**Outcomes:**

The operation was successful, and the patient made a full recovery. Follow-up imaging at 2 months confirmed complete resolution of the gastric mass.

**Lessons:**

This rare case highlights that pancreatic pseudocysts can cause major GI bleeding. Unexplained bleeding patients should be evaluated for pancreatic disease, especially with a history of pseudocysts.

## 1. Introduction

Upper gastrointestinal bleeding caused by pancreatitis is rare. Pancreatic pseudocyst is secondary to acute pancreatitis and chronic pancreatitis. If there is no obvious complication, it does not need special treatment. When the pancreatic pseudocyst erodes the artery to form a pseudoaneurysm and forms a fistula in the digestive tract at the same time, it may lead to fatal bleeding. In this paper, we report a rare case of pancreatic pseudocyst invading the gastric wall and left gastric artery branch, leading to upper gastrointestinal bleeding.

## 2. Case presentation

A 45-year-old Chinese male presented to our emergency department with recurrent melena and hematemesis for 2 weeks. On May 5, 2023, he visited a local hospital due to “melena and hematemesis.” Blood tests showed hemoglobin levels at 99 g/L, gastroscopy confirmed gastrointestinal bleeding. After conservative treatment, he was discharged. One week after post-discharge, he experienced melena again. Upon readmission, his hemoglobin level was 71 g/L. Gastroscopy indicated Dieulafoy disease, and conservative treatment was administered. Three days after admission, the patient experienced hematemesis with hemoglobin levels dropping to 43 g/L. He underwent digital subtraction angiography catheter angiography (details from the external hospital are unclear), but the cause was not identified, leading to his referral to our hospital. The patient had 2 episodes of pancreatitis in November 2022 and March 2023 and underwent gastrointestinal polypectomy in February 2023. He has no history of abdominal surgery or trauma. Physical examination showed no abnormalities.

**Figure 1.**
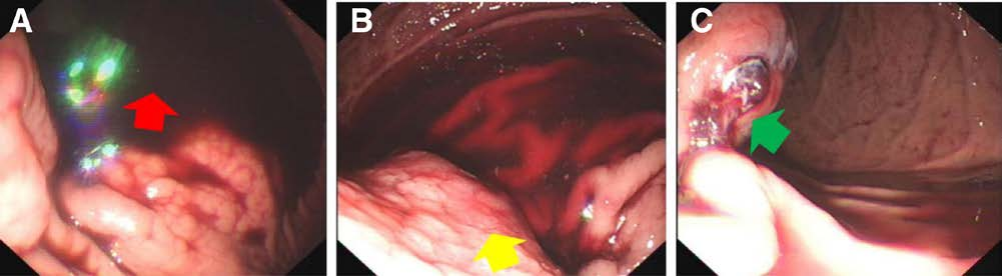
(A) The gastric fundus shows a large amount of black blood clots and dark red blood-like fluid (red arrow). (B) The gastric fundus shows a large externally compressed lesion (yellow arrow). (C) An ulcer can be seen on the surface of the raised lesion (green arrow).

**Figure 2.**
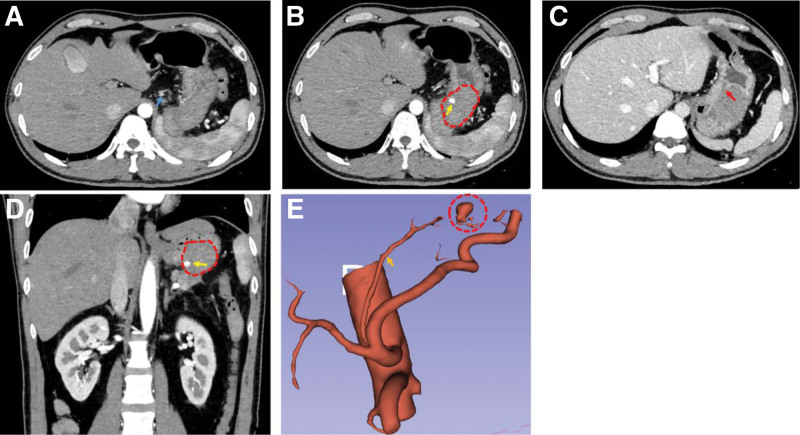
Preoperative enhanced CT of the upper abdomen. (A, B) Arterial phase in transverse view showing the left gastric artery (blue arrow), arterial aneurysm within the pseudocyst (yellow arrow), pancreatic pseudocyst (dotted circle). (C) Portal venous phase in transverse view showing discontinuity of the gastric wall (red arrow). (D) Arterial phase in coronal view showing the arterial aneurysm within the pseudocyst (yellow arrow) and the pancreatic pseudocyst (dotted circle). (E) Three-dimensional reconstruction showing the arterial aneurysm within the pseudocyst (dotted circle). CT = computed tomography.

Upon admission, the patient’s vital signs were stable. An emergency gastroscopy revealed a large amount of black blood clots and dark red fluid resembling blood in the gastric fundus. A large external compression lesion with an ulcer on its surface was observed in the gastric fundus, but no active bleeding was observed (Fig. 1). An enhanced computed tomography (CT) scan of the upper abdomen showed a slightly hyperdense mass in the gastric fundus, approximately 4.5 × 3.6 cm in size. Post-contrast, nodular enhancement was seen on the surface of the lesion, but no significant enhancement was noted within the lesion itself. The gastric wall mucosal line was discontinuous during the portal venous phase. Diagnosis: submucosal mass in the gastric fundus, suspected to be a hematoma (Fig. 2).

Through examination, the patient was diagnosed with upper gastrointestinal bleeding. The bleeding was considered to be caused by a pseudocyst resulting from chronic pancreatitis, which eroded the left gastric vessels and the gastric wall. The rupture of an aneurysm in the left gastric artery within the pseudocyst led to bleeding that entered the digestive tract through the perforated gastric wall, resulting in clinical symptoms of hematemesis and melena.

**Figure 3.**
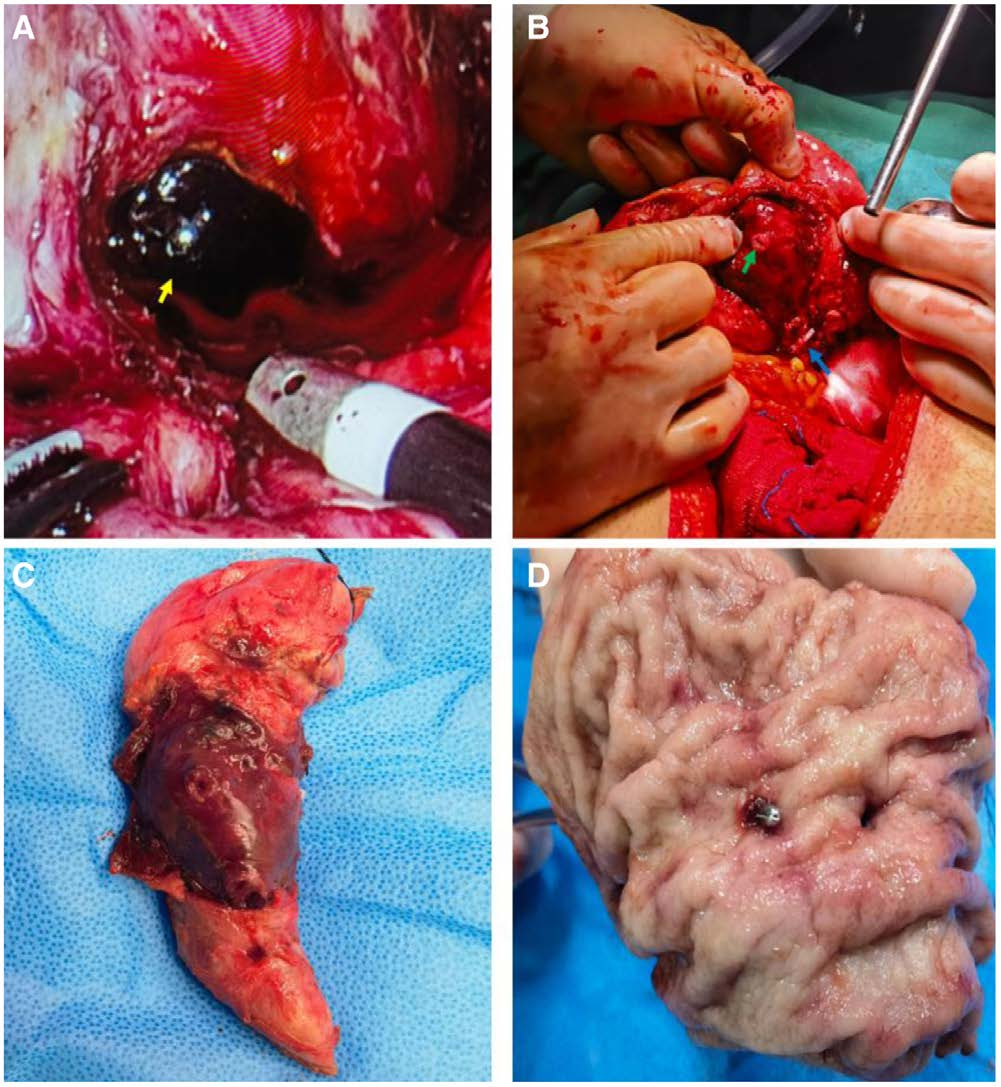
(A) After cutting the gastrocolic ligament, a cystic mass on the posterior wall of the stomach is visible, containing a large amount of blood clots (yellow arrow); active bleeding can be observed. (B) After fully mobilizing the lesser curvature side and ligating the branches of the left gastric artery (blue arrow), a perforation of the stomach wall is visible. (C) Specimen after partial gastrectomy. (D) Upon opening the specimen, 2 full-thickness perforations of the stomach wall are visible.

**Figure 4.**
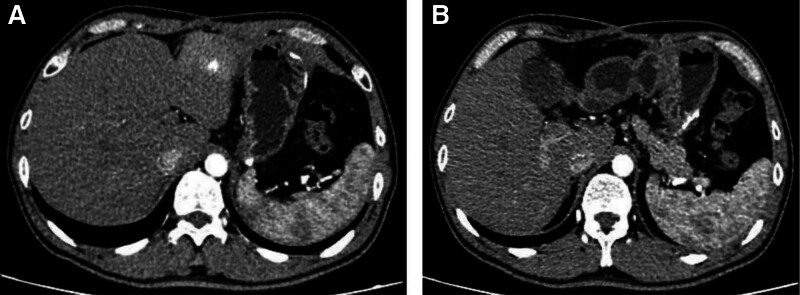
Enhanced CT scan results at the 2-month postoperative follow-up show no significant mass, with a clear outline of the pancreatic capsule. CT = computed tomography.

After identifying the cause of the upper gastrointestinal bleeding, we proceeded with surgical treatment. After separating the perigastric adhesions, a cystic mass was observed, densely adhered to the posterior gastric wall and the body and tail of the pancreas. Upon opening the cyst wall, a large amount of blood clots was found, and active bleeding from a branch of the left gastric artery was observed (Fig. 3A). The bleeding branch of the left gastric artery was ligated. After fully mobilizing the lesser curvature of the stomach, a transmural perforation of the posterior gastric wall was observed (Fig. 3B). A wedge resection of the perforated gastric wall was performed (Fig. 3C). Upon opening the resected specimen, 2 transmural perforations were seen in the posterior gastric wall (Fig. 3D). The patient recovered smoothly postoperatively without further symptoms of gastrointestinal bleeding. A follow-up 2 months after surgery showed the disappearance of the mass in the posterior gastric wall (Fig. 4).

## 3. Discussion

Pancreatic pseudocyst refers to the accumulation of fluid surrounded by non-epithelial tissue around or within the pancreas. It is a type of pancreatic cystic lesion, often secondary to late-stage acute pancreatitis or chronic pancreatitis. Broadly speaking, pancreatic pseudocysts include pancreatic pseudocysts and walled-off necrosis. The incidence of pancreatic pseudocysts is relatively high, occurring in 10% to 26% of late-stage acute pancreatitis cases and up to 20% to 40% of chronic pancreatitis cases. Improper management of pancreatic pseudocysts can lead to serious complications such as bleeding within the cyst, abdominal cavity, or digestive tract, pancreatic fistula, and intestinal fistula.

The pathophysiological cascade involves 4 key mechanisms: enzymatic degradation, dissolution of peripancreatic arteries and adjacent tissues by trypsin and elastase proteolytic activity; pseudoaneurysm vulnerability, high-flow arterial hemodynamics induces progressive wall thinning in pseudoaneurysms, predisposing to rupture; coagulopathy, pancreatic enzyme-mediated activation of the fibrinolytic system impairs hemostasis^[1]^; sinistral portal hypertension, splenic vein compression by pseudocysts may trigger left-sided portal hypertension, contributing to bleeding.^[2]^ Gastrointestinal bleeding in this context represents a dynamic pathological process.

Digestive tract bleeding caused by pancreatic cysts primarily includes 2 types: rupture of a pseudoaneurysm, where blood flows back into the pancreatic duct through the pseudocyst, causing upper gastrointestinal bleeding, also known as pseudohemobilia since it originates from the extrahepatic biliary tract^[3]^; the formation of a fistula between the pancreatic pseudocyst and the gastrointestinal tract, where after the rupture of the pseudoaneurysm, blood enters the digestive tract through the fistula, causing upper gastrointestinal bleeding.^[4]^

The most commonly affected arteries in pseudoaneurysm rupture due to chronic pancreatitis include the splenic artery (60–65%),^[4]^ the gastroduodenal artery (20–25%), and the pancreaticoduodenal artery (10–15%).^[5]^

In cases of acute or chronic pancreatitis accompanied by gastrointestinal bleeding, the possibility of pancreatic pseudocyst erosion into the digestive tract and blood vessels should be ruled out. The primary diagnostic modalities for exclusion include contrast-enhanced CT of the upper abdomen and digital subtraction angiography (DSA). In this case, acute hemorrhage is triggered when a pseudocyst erodes the left gastric artery and the gastric wall, leading to the accumulation of coagulated blood within the cyst cavity. Bleeding ceases as the intracystic pressure exceeds the arterial pressure. Subsequently, the intracystic pressure is reduced due to clot degradation by pancreatic enzymes, which can result in recurrent gastrointestinal hemorrhage. Consequently, patients are prone to experience repeated episodes of bleeding.

As DSA requires arterial puncture, the procedure carries risks such as puncture site hematoma, vascular injury, thrombosis, or intestinal ischemic necrosis. Although DSA holds significant advantages for diagnosis during active bleeding episodes, its application in some primary care hospitals is limited. This is due to a lack of necessary equipment and physicians qualified to perform the procedure. Furthermore, in such complex cases, DSA can yield negative results, potentially leading to misdiagnosis.

In contrast, contrast-enhanced CT of the upper abdomen offers the advantages of straightforward operation and high reproducibility. Its primary diagnostic findings include the retention of contrast within the cyst cavity and the clear visualization of an extrapancreatic mass with discontinuity of the digestive tract. Studies have indicated that the combination of abdominal contrast-enhanced CT and DSA can significantly reduce the rate of negative angiographic findings and minimize misdiagnosis.^[6]^

Therefore, for patients suspected of arterial hemorrhage secondary to a pancreatic pseudocyst, contrast-enhanced abdominal CT is recommended as a 1st-line diagnostic method. If resources permit, it can be combined with DSA for further definitive diagnosis.

## 4. Conclusion

The diagnosis of gastrointestinal bleeding caused by pancreatic pseudocysts is relatively difficult. Currently, clinical diagnosis of this disease mainly relies on imaging examinations, including CT, magnetic resonance imaging, and endoscopic ultrasonography.^[7]^ The main causes of massive gastrointestinal bleeding due to pancreatic pseudocysts include: erosion of surrounding arteries leading to hemorrhage within the pancreatic duct; formation of a fistula between the eroded surrounding arteries and a hollow viscus while the cyst erodes them.^[8,9]^ The management of massive upper gastrointestinal bleeding associated with pancreatic pseudocysts primarily involves interventional and surgical treatments, surgery being the optimal therapeutic approach. Surgery not only treats the bleeding but also addresses the pseudocyst itself.

## Author contributions

**Investigation:** Shifeng Teng.

**Supervision:** Shifeng Teng.

**Writing – original draft:** Shifeng Teng.

**Writing – review & editing:** Shifeng Teng, Minghao Huang.
